# Chimeric adenoviral (Ad5.F35) and listeria vector prime-boost immunization is safe and effective for cancer immunotherapy

**DOI:** 10.1038/s41541-022-00483-z

**Published:** 2022-06-23

**Authors:** John C. Flickinger, Ross E. Staudt, Jagmohan Singh, Robert D. Carlson, Joshua R. Barton, Trevor R. Baybutt, Jeffrey A. Rappaport, Alicja Zalewski, Amanda Pattison, Scott A. Waldman, Adam E. Snook

**Affiliations:** 1 Department of Pharmacology, Physiology, & Cancer Biology, Sidney Kimmel Medical College, Thomas Jefferson University, Philadelphia, PA 19107 USA; 2 Department of Surgery, Sidney Kimmel Medical College, Thomas Jefferson University, Philadelphia, PA 19107 USA; 3 Sidney Kimmel Cancer Center, Jefferson Health, Philadelphia, PA 19107 USA

**Keywords:** Preclinical research, Vaccines, Tumour immunology, Gastrointestinal cancer, Cancer immunotherapy

## Abstract

Strategies to augment immunity to self/neoantigens expressed by cancers are urgently needed to expand the proportion of patients benefiting from immunotherapy, particularly for GI cancers where only a fraction of patients respond to immunotherapies. However, current vaccine strategies are limited by poor immunogenicity, pre-existing vector-specific immunity, and vaccine-induced vector-specific immunity. Here, we examined a prime-boost strategy using a chimeric adenoviral vector (Ad5.F35) that resists pre-existing immunity followed by recombinant *Listeria monocytogenes* (Lm) to amplify immunity to the GI cancer antigen GUCY2C. This previously unexplored combination enhanced the quantity, avidity, polyfunctionality, and antitumor efficacy of GUCY2C-specific effector CD8^+^ T cells, without toxicity in any tissue, including GUCY2C-expressing intestines and brain. Importantly, this combination was partially resistant to pre-existing immunity to Ad5 which is endemic in human populations and vector-specific immunity did not limit the ability of multiple Lm administrations to repeatedly enhance GUCY2C-specific responses. Broadly, these findings suggest that cancer patient immunizations targeting self/neoantigens, as well as immunizations for difficult infectious diseases (HIV, malaria, etc), may be most successful using a combination of Ad5.F35-based priming, followed by Lm-based boosting. More specifically, Lm-GUCY2C may be utilized to amplify GUCY2C-specific immunity in patients receiving adenovirus-based GUCY2C vaccines currently in clinical trials to prevent or treat recurrent GI cancer.

## Introduction

Immune checkpoint blocking (ICB) therapy has revolutionized cancer treatment and established immunotherapy as a pillar of cancer management. However, success with ICB therapy is predicated upon a high tumor-mutational burden (TMB) and the presence of immune cell infiltration^1–3^. Thus, ICB therapy is largely ineffective for the vast majority of immunologically ‘cold’ tumors that lack targetable tumor-specific mutations and/or sufficient levels of tumor-infiltrating lymphocytes. Indeed, a recent estimate suggests that ICB therapy is effective for only 12.5% of patients^4^. Thus, more effective immunotherapeutic strategies to treat immunologically ‘cold’ tumors are urgently needed.

Recently, vaccines have re-emerged as a viable option to treat or prevent recurrent cancer. In the context of an immunologically ‘cold’ tumor, cancer vaccines can prime *de novo* tumor-reactive T cells and expand existing tumor-specific responses leading to enhanced tumor immune cell infiltration^5^ conferring antitumor immunity alone^6^ or promoting ICB responsiveness^7^. While promising, the success of cancer vaccines has, in part, been hindered by a lack of suitable methods to produce high quality tumor-specific immunity in patients. Despite the creation of numerous vaccination platforms (bacterial, viral, nucleic acid, etc.), each method carries distinct limitations and there is no consensus as to an optimal cancer vaccination method^8^. Bacterial and viral vectors are often popular strategies due to their ability to potently stimulate numerous inflammatory pathways. However, the efficacy of bacterial-based vaccines in clinical trials to date has been disappointing^9^ and some platforms only weakly prime tumor-specific CD8^+^ T-cell responses^10,11^. While viral vectors are often effective at priming responses, their efficacy is reduced in patients with pre-existing vector-specific immunity^7^ and by the induction of vaccine-induced vector-specific antibodies that limit repeated immunizations^12^. In contrast, DNA-based and peptide-based strategies, while not limited by vector-specific immunity, have historically demonstrated poor immunogenicity in clinical trials^13–15^. While RNA vaccines have recently demonstrated success in vaccinating against infectious diseases^16^, their effectiveness in the context of cancer is unclear^17,18^.

Recently, we demonstrated that the chimeric adenoviral vector, Ad5.F35, is not limited by endemic Ad5-specific immunity and may produce immune responses in ~90% of the human population^19^. While effective at priming tumor-specific CD8^+^ T-cell responses, optimal immunity towards an antigen often requires multiple antigenic encounters^20^ and Ad5.F35 is expected to induce vector-specific antibodies upon vaccination, limiting the efficacy of repeated immunization. In contrast to Ad5.F35 which is limited to a single injection, the Gram-positive bacterium *Listeria monocytogenes* (Lm) is a cancer vaccine platform that is not neutralized by vector-specific antibodies, permitting repeated immunization^21,22^. Moreover, Lm activates numerous aspects of the innate and adaptive immune system^23^ and can remodel immunosuppressive tumor microenvironments^24,25^. While Lm vaccines have demonstrated poor CD8^+^ T-cell priming for some tumor antigens^10,11^, we hypothesized that its resistance to vector-specific immunity may make it an ideal platform for prime-boost strategies following Ad5.F35-based vaccines.

This strategy could overcome the limitations of ICB in colorectal cancer (CRC) to treat or prevent disease recurrence following conventional therapies. Indeed, ICB therapy is approved for only a small subset (~15%) of CRC patients whose tumors have high TMB^26^. Thus, effective immunotherapeutics to treat the remaining (~85%) CRC patients are lacking. In this context, the intestinal receptor and tumor-associated antigen guanylyl cyclase C (GUCY2C) is an emerging immunotherapeutic target in CRC. In contrast to neoantigens which may be unpredictable and lacking across TMB-low tumors^27^, GUCY2C protein is present across nearly all CRC tumors^28,29^, and expression among endogenous tissues is limited^30,31^. GUCY2C-directed vaccines are being developed as a secondary preventive measure to protect against metastatic CRC in high-risk patients following conventional surgical and/or chemo/targeted therapies^32^. Recently, a phase I trial testing a GUCY2C vaccine safely generated GUCY2C-specific immune responses in patients^33^, and an ongoing phase IIa trial is testing a chimeric Ad5.F35-based GUCY2C vaccine (NCT04111172). Thus, we explore here an Ad5.F35-based GUCY2C vaccine in combination with a recombinant strain of Lm secreting GUCY2C (Lm-GUCY2C). We evaluated the immunogenicity of Ad5.F35 + Lm vaccination regimens and demonstrate that optimal GUCY2C-specific immunity (T-cell quantity, avidity, and polyfunctionality) is achieved using a heterologous prime-boost strategy in which Ad5.F35 is used to ‘prime’ responses for boosting with Lm. Moreover, this strategy enhances long-term memory responses and is only partially limited by pre-existing immunity to Ad5 or Lm. Finally, we demonstrate that this strategy does not elicit toxicity and may be rapidly translated to future clinical trials.

## Results

### Lm-GUCY2C vaccine design

**Fig. 1:**
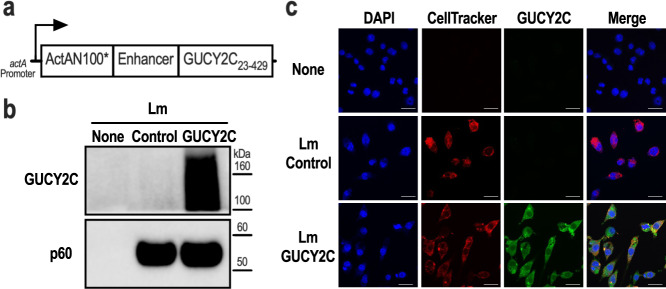
Construction of Lm-GUCY2C. **a** Lm-GUCY2C secretes a fusion protein comprised of ActAN100*, an enhancer sequence, and mouse GUCY2C_23-429_ under the control of the *actA* promoter. **b**, **c** J774A.1 macrophages were uninfected or infected with Lm-Control or Lm-GUCY2C at a 10:1 MOI for 6 h at 37 °C. GUCY2C fusion protein was detected by (**b**) western blot and (**c**) immunofluorescence. Scale bars in (**c**) are 25 μm. Full western plots are shown in Supplementary Fig. S7.

The extracellular domain of mouse GUCY2C (GUCY2C_23-429_) was codon-optimized for *Listeria monocytogenes* using Java Codon Adaptation Tool^34^ and synthesized downstream of the *actA* promoter, a modified version of the first 100 amino acids of ActA (ActAN100*)^35^, and an enhancer sequence (Fig. 1a). The resulting sequence was cloned into the pPL2^36^ integration vector and stably integrated into the genome of the live attenuated double-deleted Lm strain Δ*actA*Δ*inlB*^37^. Secretion of the ActA-GUCY2C fusion protein was confirmed in the J774A.1 macrophage cell line 6 h after infection with Lm-GUCY2C or control Lm strain by western blot (Fig. 1b) and immunofluorescence (Fig. 1c) staining with a GUCY2C-specific antibody.

### Heterologous Ad-GUCY2C+Lm-GUCY2C immunization enhances GUCY2C-specific CD8^+^ T-cell responses and antitumor immunity

**Fig. 2:**
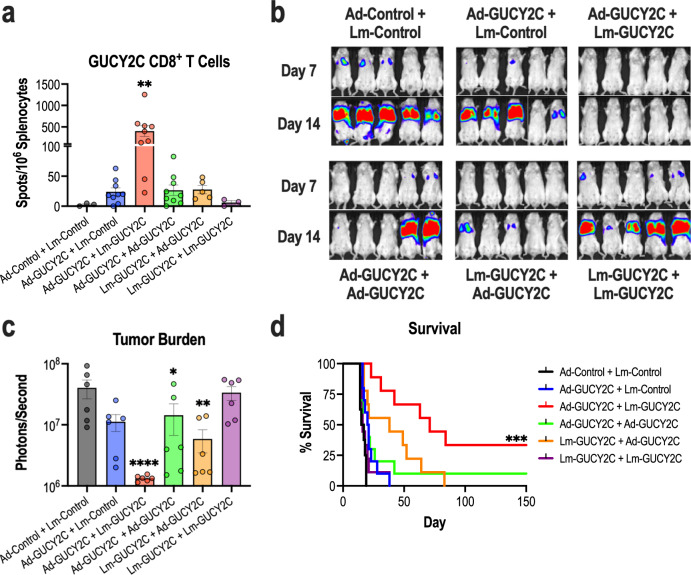
Heterologous Ad5.F35+Lm immunization enhances GUCY2C-specific CD8^+^ T-cell responses and antitumor immunity. **a**–**d** BALB/cJ mice (*n* = 3–9/group) were immunized with a ‘priming’ immunization on day 0 and a ‘boosting’ immunization on day 21 utilizing homologous or heterologous combinations of GUCY2C-expressing and control vaccines. GUCY2C or control adenovirus vaccines were administered intramuscularly (i.m.) at 10^10^ vp and GUCY2C or control Lm vaccines were administered intravenously (i.v.) at 5 × 10^6^ CFU. Six days after the final immunization, spleens were collected and splenocytes were stimulated with GUCY2C_254-262_ peptide to quantify GUCY2C-specific CD8^+^ T cells by IFNγ ELISpot (**a**) or mice were challenged i.v. with 5 × 10^5^ CT26 colorectal cancer cells expressing GUCY2C and firefly luciferase (**b**–**d**). **b** On days 7 and 14 post-tumor challenge, mice were injected with D-luciferin substrate and imaged. **c** Day 7 tumor burden was quantified by imaging. **d** Survival was monitored throughout the experiment. GUCY2C-specific CD8^+^ T-cell counts (**a**) and tumor-burden (**c**) were analyzed by one-way ANOVA compared to control immunization with Dunnett’s test to correct for multiple comparisons. Survival was analyzed by the Mantel-Cox log-rank test with all immunized groups compared to control immunization using the Bonferroni method to correct for multiple comparisons (**d**). Error bars indicate mean +/− SEM.

Following in vitro confirmation of GUCY2C fusion protein expression by Lm-GUCY2C, we next sought to identify an optimal GUCY2C immunization regimen. Thus, Lm-GUCY2C vaccine was tested in combination with the chimeric adenovirus-based (Ad5.F35) vaccine against GUCY2C (“Ad-GUCY2C”) currently in phase II clinical testing (NCT04111172). GUCY2C immunogenicity and antitumor immunity were evaluated utilizing a prime-boost strategy in which vaccines were administered 21 days apart as homologous or heterologous vaccinations. Notably, the heterologous immunization regimen utilizing Ad-GUCY2C to ‘prime’ GUCY2C-specific immune responses followed by Lm-GUCY2C to ‘boost’ those responses generated significantly higher GUCY2C-specific effector CD8^+^ T-cell responses quantified by IFNγ ELISpot compared to all other immunization regimens (Fig. 2a). Similarly, in a model of recurrent colorectal cancer metastases, Ad-GUCY2C followed by Lm-GUCY2C immunization significantly reduced metastatic tumor burden (Fig. 2b–c) and increased survival (Fig. 2d) over other combinations. Importantly, the order of immunization was essential for optimal GUCY2C immunity, with Ad-GUCY2C + Lm-GUCY2C inducing a >15-fold increase in GUCY2C-specific CD8^+^ T-cells and maximally extending median survival (71 vs. 38 days) compared to Lm-GUCY2C + Ad-GUCY2C vaccination. However, in a therapeutic model, an abbreviated Ad-GUCY2C + Lm-GUCY2C prime-boost regimen (7-day interval) failed to stop disease progression (Supplementary Fig. S1), highlighting the need for more aggressive immunotherapeutic strategies in that setting, such as CAR-T cell therapy^38,39^. Next, since memory T-cells are expected to be vital for long-term protection against recurrent cancer, the ability of Lm-GUCY2C to produce long-lasting immunity following adenovirus-based vaccination was determined (Supplementary Fig. S2A). Sixty-three days after completing immunizations, GUCY2C-specific CD8^+^ T-cell memory assessed by ELISpot or tumor challenge demonstrated that boosting with Lm-GUCY2C substantially enhanced GUCY2C-specific CD8^+^ T-cell counts (Supplementary Fig. S2B), reduced metastatic tumor burden (Supplementary Fig. S2C), and improved overall survival (25 vs >90 days; Supplementary Fig. S2D) compared to mice that received only adenovirus-based priming. Finally, to determine the ability of Lm-GUCY2C to boost GUCY2C-specific memory responses long after an initial priming vaccination, mice were boosted with control or GUCY2C Lm >100 days after initial priming with Ad-GUCY2C (Supplementary Fig. S3). As expected, mice boosted with Lm-GUCY2C exhibited robust secondary expansion of GUCY2C-specific memory CD8^+^ T cells, suggesting that Lm-GUCY2C could be utilized long after an initial priming vaccination (Supplementary Fig. S3).

### Prior Ad5, but not Lm, exposure limits Ad5.F35+Lm immunization regimens

**Fig. 3:**
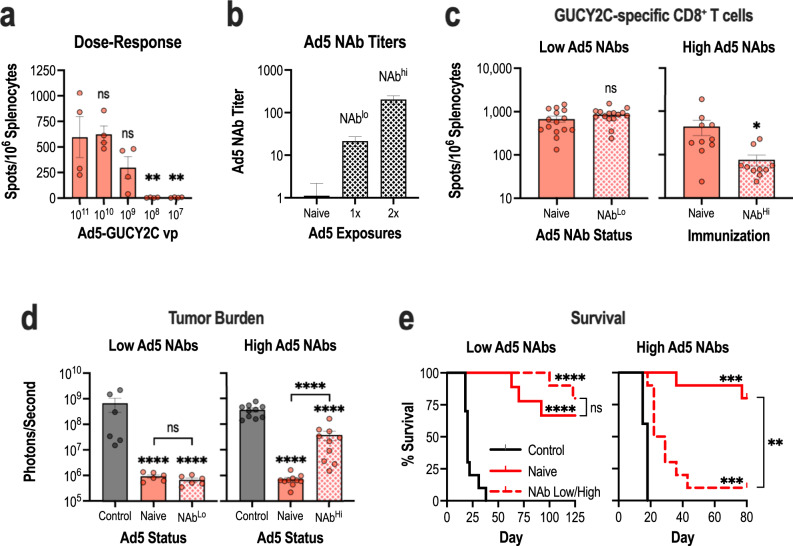
High levels of pre-existing Ad5 immunity limits Ad5.F35+Lm efficacy. **a** BALB/cJ (*n* = 4/group) mice were immunized with decreasing doses (10^11^ vp - 10^7^ vp) of Ad5.F35-based GUCY2C (Ad-GUCY2C) vaccine on day 0 and boosted with 5 × 10^6^ CFU of Lm-GUCY2C on day 21. Six days after the final vaccination, GUCY2C-specific CD8^+^ T-cell responses were quantified by IFNγ ELISpot. **b**–**e** BALB/cJ mice were intranasally infected once or twice (28-day interval) with 10^10^ vp of Ad5-GFP or PBS as a control. **b** Twenty-eight days following Ad5 exposure(s), Ad5-specific NAbs were confirmed in sera (*n* = 5–20/group) prior to GUCY2C vaccination in (**c**–**e**). **c–e** Naive or Ad5-exposed mice were then immunized with 10^10^ vp Ad-GUCY2C and boosted with 5 × 10^6^ CFU Lm-GUCY2C as in Fig. 2 (controls received control Ad and Lm). Six days after the final vaccination, 10–15 mice/group were euthanized to evaluate GUCY2C-specific CD8^+^ T-cell responses (**c**), and the remaining 6–10 mice/group were challenged with 5 × 10^5^ CT26 colorectal cancer cells expressing GUCY2C and firefly luciferase (**d**–**e**). **d** On day 11 (NAb^hi^ groups) or 14 (NAb^lo^ groups) following tumor challenge, tumor burden was quantified. **e** Survival was monitored throughout the experiment. GUCY2C-specific CD8^+^ T-cell counts in (**a**) and tumor burden (**d**) were analyzed by one-way ANOVA compared to control immunization (**a**) or all groups (**d**) with Dunnett’s test to correct for multiple comparisons. GUCY2C-specific CD8^+^ T-cell counts in (**c**) were compared by a two-sided *T*-test. Survival was analyzed by the Mantel-Cox log-rank test with indicated comparisons using the Bonferroni method to correct for multiple comparisons (**e**). Error bars indicate mean +/− SEM.

A known limitation of viral-based vaccines is the ability of vector-specific immunity to interfere with the induction of responses to the target transgene. Specifically, neutralizing antibodies (NAbs) against the common adenovirus serotype, Ad5, are detectable in >70% of healthy donors and blunt the efficacy of Ad5-based vaccines^40–42^. Although chimeric adenoviruses, such as Ad5.F35, are less susceptible to neutralization associated with Ad5-specific NAbs, Ad5.F35-induced GUCY2C-specific immune responses are partially reduced in the context of prior Ad5 exposure^19^, which may extend to prime-boost regimens based on Ad5.F35 and Lm. In contrast to adenovirus, Lm infection in humans does not result in NAbs against Lm and prior exposure does not limit immune responses to the target antigen^21,22^. Consistent with these observations, we found that prior Lm exposure did not diminish boosting with Lm-GUCY2C (Supplementary Fig. S4A–B). Moreover, Lm-GUCY2C could be administered repeatedly to enhance GUCY2C-specific CD8^+^ T-cell responses (Supplementary Fig. S4C). Thus, we focused on the impact of prior Ad5 exposure on the Ad5.F35 + Lm vaccination approach. First, Lm-GUCY2C boosted responses following low priming doses of Ad5.F35, which could result from antibody-mediated neutralization (Fig. 3A). Indeed, Lm-GUCY2C boosted GUCY2C-specific CD8^+^ T-cell responses even at priming doses of 10^9^ vp, 10-fold lower than the standard priming dose of 10^10^ vp (Fig. 3A). Next, to mimic pre-existing immunity to Ad5 in vivo, mice were exposed intranasally to PBS (naive) or 10^10^ vp of Ad5-GFP prior to the start of an Ad5.F35 + Lm prime-boost. One pre-exposure to Ad5 induces low Ad5 NAbs (titer of 20), while two pre-exposures induce high Ad5 NAbs (titer >200)^19^. As expected, one or two Ad5 exposures induced NAb titers of 20 and >200, respectively, in mice at the time of Ad5.F35 + Lm vaccination (Fig. 3b). GUCY2C-specific CD8^+^ T-cell responses were equivalent following Ad5.F35 + Lm immunization of naive and Ad5 NAb^lo^ mice, however, responses were reduced ~10-fold in Ad5 NAb^hi^ animals (Fig. 3c). For context, that level of response is comparable to a single immunization of naive mice with Ad5.F35-GUCY2C (Fig. 2a). Despite the reduced magnitude of the CD8^+^ T-cell response in Ad5 NAb^hi^ animals, they retained some antitumor efficacy (Fig. 3d). Using the recurrence model employed in Fig. 2, tumor burden in naive and NAb^lo^ mice immunized with Ad5.F35 + Lm was equivalent and low; however, while tumor burden was lower in NAb^hi^ mice than controls, it was higher than Ad5-naive animals (Fig. 3d). There was a similar impact on survival. Naive and NAb^lo^ animals had comparable survival times (Fig. 3e). While NAb^hi^ animals immunized with Ad5.F35 + Lm were protected compared to control animals, their survival was reduced compared to naive mice that received Ad5.F35 + Lm (Fig. 3e). Thus, these data suggest that high levels of Ad5 immunity indeed impact Ad5.F35 + Lm efficacy. However, given (1) the partial efficacy of Ad5.F35 + Lm despite high Ad5 immunity (Fig. 3d–e) and (2) the ability of repeated Lm exposures to amplify responses (Supplementary Fig. S4C), even long after initial Ad5.F35 priming (Supplementary Fig. S3), this may be overcome in patients by repeated Lm-GUCY2C boosts, as is typically done with Lm-based vaccines in patients^21,23,43^.

### Qualitative changes in the CD8^+^ T-cell pool following prime-boost vaccination

**Fig. 4:**
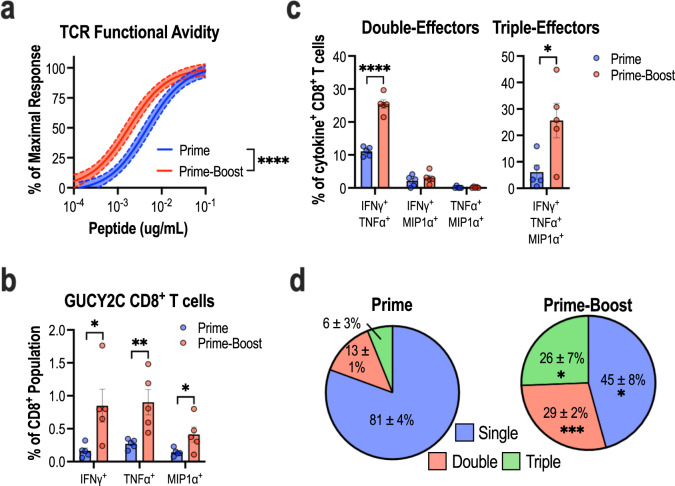
Ad5.F35+Lm enhances the avidity and polyfunctionality of the GUCY2C-specific CD8^+^ T-cell pool. BALB/cJ mice (*n* = 5–8/group) were immunized using Ad-GUCY2C (prime) or Ad-GUCY2C + Lm-GUCY2C (prime-boost) vaccination regimens. At the peak effector response, (14 days following prime and 6 days following prime-boost vaccination), splenocytes were collected and stimulated with GUCY2C_254-262_ peptide to assess GUCY2C-specific CD8^+^ T-cell avidity quantified by IFNγ ELISpot (**a**) and polyfunctionality quantified by flow cytometry (**b**–**d**). **a** Non-linear regression (solid line) of GUCY2C-specific CD8^+^ T cell avidity is depicted with 95% confidence intervals (dashed lines). **b** The proportion of live CD8^+^ T cells staining positive for IFNγ, TNFα, or MIP1α cytokines after stimulation with GUCY2C_254-262_ peptide is shown**. c**, **d** Of cytokine^+^ CD8^+^ T cells, the percentage of cells staining positive for two and three cytokine-markers (**c**) as well as overall polyfunctionality (**d**) are shown. Polyfunctionality comparisons were made using two-sided *T*-tests comparing prime vs prime-boost with Holm-Sidak correction for multiple comparisons. Error bars indicate mean +/− SEM. Representative FACS plots are shown in Supplementary Fig. S8.

In addition to expanding the quantity of antigen-specific T cells, previous studies have demonstrated that various prime-boost immunizations impact the quality of antigen-specific T cells^44–46^. Thus, the impact of Ad5.F35 + Lm on the GUCY2C-specific CD8^+^ T-cell pool was determined by comparing the avidity and polyfunctionality of GUCY2C-specific CD8^+^ T cells at the peak effector response following prime-only and prime-boost vaccination. The avidity of the GUCY2C-specific CD8^+^ T-cell pool was determined by pulsing splenocytes from immunized mice with decreasing concentrations of GUCY2C_254-262_ peptide to define the EC_50_ of this response. Compared to mice immunized with a priming immunization alone, the EC_50_ of mice immunized with prime-boost was shifted ~2.5-fold (4.6 vs. 1.8 ng/mL, *P* < 0.0001), suggesting an enrichment of high-avidity GUCY2C-specific T cells in these mice (Fig. 4a). Flow cytometry was employed to assess the polyfunctionality of GUCY2C-specific CD8^+^ T cells after prime-only and prime-boost vaccination. Consistent with ELISpot experiments, prime-boost immunization significantly enhanced the proportion of CD8^+^ T cells with GUCY2C-specificity compared to priming alone indicated by an increased percentage of CD8^+^ T cells producing IFNγ (Fig. 4b), as well as the effector cytokines TNFα and MIP1α after stimulation with GUCY2C_254-262_ peptide (Fig. 4b). Among cytokine-producing GUCY2C-specific CD8^+^ T cells, the percentage of cells simultaneously displaying two or three effector functions was significantly enriched in the prime-boost group compared to the priming-only group (Fig. 4c). Priming alone induced GUCY2C-specific CD8^+^ T cells with a predominantly single effector function (81 ± 4%; Fig. 4d). In contrast, prime-boost immunization predominately generated GUCY2C-specific CD8^+^ T cells displaying multiple effector functions with only a minority of these cells exhibiting a single effector function (45 ± 8%; Fig. 4d). Evaluation of plasma cytokines revealed only minor differences between animals receiving control Ad5.F35 + Lm prime-boost and those receiving GUCY2C Ad5.F35 + Lm prime-boost (Supplementary Fig. S5). Thus, in addition to amplifying the quantity of GUCY2C-specific CD8^+^ T cells, Lm-GUCY2C boosting induced qualitative enhancements in the avidity and polyfunctionality of the GUCY2C-specific CD8^+^ T-cell pool.

### Heterologous prime-boost immunization does not elicit toxicity

**Fig. 5:**
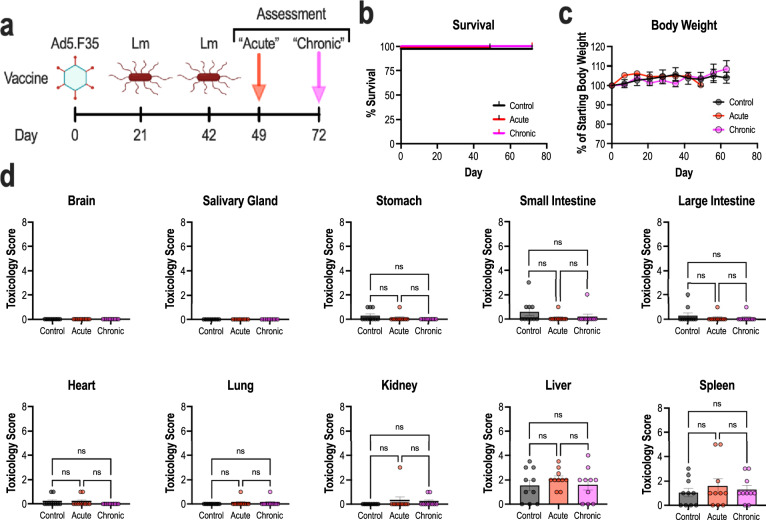
Heterologous prime-boost immunization does not induce toxicity. **a**–**d** BALB/cJ mice (*n* = 10/group) were separated into three different cohorts to assess toxicity. A control group received PBS on day 0, 21, and 42 and was sacrificed on day 72. “Acute” and “chronic” cohorts received Ad-GUCY2C (Ad5.F35) on day 0 and Lm-GUCY2C on days 21 and 42 and were sacrificed on days 49 and 72, respectively. Survival (**b**) and body weight (**c**) were monitored throughout the study and demonstrated no change from control. **d** Organs from mice were collected, fixed in formalin, and paraffin embedded. Slides were cut, H&E stained, and scored by a blinded pathologist. Survival (**b**) was analyzed by the Mantel-Cox log rank. Body weights (**c**) were analyzed by two-way ANOVA (no significant difference between groups). Histology (**d**) was analyzed by one-way ANOVA. Error bars indicate mean +/− SEM.

Previously, GUCY2C-specific vaccines induced systemic anti-tumor immunity without invoking autoimmunity towards endogenous GUCY2C-expressing tissues^19,47–52^. Given increases in the quantity, avidity, and polyfunctionality of the GUCY2C-specific CD8^+^ T-cell pool following Lm-GUCY2C-boosting, we wanted to characterize the safety of this immunization regimen. Thus, we employed two vaccination and assessment schedules to elucidate potential toxicity. Mice were primed with 10^10^ vp Ad5.F35-GUCY2C on day 0, followed by two boosts of 5 × 10^6^ CFU Lm-GUCY2C on days 21 and 42 (Fig. 5a). Seven and thirty days following final immunization (days 49 and 72 overall), mice were euthanized, and tissues were analyzed by histopathology for assessment of acute and chronic toxicity, respectively. An additional control cohort received PBS on all immunization days. No signs of toxicity in acute or chronic cohorts were detected by in-life observations, survival (Fig. 5b), or body weight (Fig. 5c) compared to the control group receiving saline only. Similarly, histopathological scoring by a blinded pathologist revealed no differences in inflammation or tissue damage (Fig. 5d) in GUCY2C-expressing tissues (small intestine, colon, brain) and GUCY2C-devoid tissues (salivary gland, stomach, heart, lung, kidney, and liver). Moreover, plasma cytokine (Supplementary Fig. S5) and clinical chemistry profiles (Supplementary Fig. S6) revealed no vector-induced or GUCY2C-specific cytokine-release syndrome (CRS) or toxicity, respectively. Collectively, these data suggest that a GUCY2C-targeted vaccination strategy employing Ad5.F35 + Lm prime-boost substantially enhances GUCY2C-specific immunity and antitumor efficacy (Figs. 2–4) without collateral autoimmunity targeting endogenous GUCY2C-expressing tissues (Fig. 5).

## Discussion

Effective cancer immunotherapeutics to date rely on their ability to enhance endogenous tumor-reactive T cells (ICB) or deliver exogenously engineered T cells (CAR-T cell therapies)^53^. While ICB therapy can accomplish this in some malignancies, its efficacy is dependent on a high TMB and pre-existing immune cell infiltration into the tumor^1–3^. Thus, ICB therapy has largely been ineffective in treating TMB-low and immunologically “cold” tumors, such as microsatellite-stable (MSS) colorectal and pancreatic cancers^2^. In this context, cancer vaccines have re-emerged as an attractive method to stimulate tumor-reactive T cells alone or in combination with ICB^7^. However, the success of cancer vaccines has been impeded, in part, by a lack of vaccination methods to reliably produce robust T-cell responses to tumor-associated antigens. Here, we identified a highly effective prime-boost immunization regimen utilizing the chimeric adenovirus, Ad5.F35, and the bacterium, Lm. Indeed, this regimen is highly immunogenic and produces potent antitumor immunity towards the colorectal tumor-associated antigen GUCY2C, without toxicity.

The induction of protective cellular immune responses often requires multiple antigenic encounters^20^. However, repeated vaccination utilizing the same vector to deliver a vaccine antigen is often limited by vector-specific immunity. Specifically, for virus-based vaccines such as Ad5, efficacy is reduced in individuals carrying Ad5-specific NAbs which are induced by prior natural infection with Ad5 or previous vaccination with Ad5-based vectors^40,41^. While, chimeric adenoviruses utilizing rare adenoviral serotypes, such as Ad5.F35, may partially overcome this limitation to induce immune responses in a high proportion of the population (~90%)^19^, they cannot overcome the limitations of vector-induced immunity following vaccination. In contrast, the bacterium Lm is not neutralized by Lm-specific antibodies and is immunogenic in the context of prior Lm exposure^21,22^. Despite this, the majority of Lm vaccines poorly prime CD8^+^ T-cell responses towards tumor-associated antigens^10,11^. Thus, we hypothesized that a novel combination of vectors in which Ad5.F35 is utilized to prime immune responses against a tumor antigen followed by Lm to boost these responses could produce a highly effective vaccination regimen—while regimens composed of Lm priming and adenovirus boosting have been described^54,55^, no literature exists exploring any regimens composed of adenovirus priming and Lm boosting. Indeed, in the context of GUCY2C-targeted vaccines, heterologous prime-boost immunization (Ad5.F35 + Lm) elicited superior antitumor immunity compared to homologous immunization with either vector (Fig. 2). Importantly, while Ad5 has limited activity in >50% of the human population^33^, this immunization regimen likely could be useful across human populations, since Ad5.F35 + Lm vaccination was effective in not only naive mice, but also in mice previously exposed to Ad5 or Lm (Fig. 3 and Supplementary Fig. S4), though high Ad5-specific immunity did have a negative impact on Ad5.F35 + Lm efficacy. Moreover, due to the minimal impact of Lm-specific immunity on Lm vaccine-induced responses^21,22^, our studies (Supplementary Fig. S4) suggest that Lm-based vaccines can be utilized repeatedly as a booster to regularly elevate immunity to therapeutic levels in patients with high pre-existing immunity to Ad5 or at-risk patients.

Heterologous prime-boost vaccination (Ad5.F35 + Lm) against GUCY2C was associated with substantial changes in the GUCY2C-specific T-cell population. Notably, this Ad5.F35 + Lm combination generated significantly higher quantities of GUCY2C-specific effector CD8^+^ T-cells compared to other combinations (Fig. 2a). Further, qualitative differences in T-cell responses also may impact vaccine efficacy. Indeed, T cells with high avidity or multiple effector cytokine functions more effectively eliminate cancer cells and viral infections^56–58^. Here, we found that Ad5.F35 + Lm vaccination produced GUCY2C-specific CD8^+^ T cells with increased avidity (Fig. 4a). Moreover, boosting with Lm-GUCY2C impacted the polyfunctionality of the GUCY2C-specific CD8^+^ T-cell pool, shifting it from a predominately unifunctional (1 cytokine) T-cell population to a population in which multifunctional T cells predominated (Fig. 4b–d). Based on these experiments, it is unclear to what extent the high-avidity T-cell population overlaps with the polyfunctional T-cell population. Thus, it is unknown whether Lm-GUCY2C directly converts unifunctional T cells into polyfunctional T cells or if it selectively induces secondary expansion of high avidity T cells that already display polyfunctionality. The avidity of cancer-specific CD8^+^ T cells may be coupled to their polyfunctionality^59^, supporting the possibility that Lm-GUCY2C induces selective secondary expansion of high-avidity T cells with enhanced functionality and antitumor efficacy. Further studies are required to delineate between these mechanisms.

While GUCY2C-expressing Lm stimulated robust expansion of GUCY2C-specific CD8^+^ T cells following priming with an established adenoviral vector^19^, Lm-GUCY2C alone was ineffective as single-agent therapy. Indeed, homologous Lm-GUCY2C vaccination failed to generate GUCY2C-specific CD8^+^ T cells by ELISpot (Fig. 2a) or antitumor efficacy (Fig. 2b–d). Interestingly, studies exploring Lm vaccines as single-agent therapies have reported mixed success. Lm vaccines against the tumor antigens HER2^60^ and PSA (prostate-specific antigen)^61^ generate potent antitumor immunity, while Lm vaccines against other tumor antigens including PAP (prostatic acid phosphatase)^10^ and mesothelin^43^ exhibited limited immunogenicity. Anti-GITR^62^ and anti-PD-1^63,64^ antibodies enhanced the antitumor efficacy of Lm vaccines, suggesting that tolerance or immunosuppressive mechanism may impede Lm vaccine efficacy. Here, immunodominance mechanisms appear to be the limiting factor for Lm-GUCY2C priming^65^. The GUCY2C_254-262_ epitope has a low affinity for H-2K^d^ and high-affinity Lm epitopes outcompete GUCY2C_254-262_, resulting in no priming of GUCY2C-specific responses^65^. Additional studies may better define mechanisms underlying the poor efficacy of Lm vaccines targeting GUCY2C and other cancer antigens, to enhance their effectiveness.

In the context of an ongoing clinical trial testing the first Ad5.F35-based vaccine in the adjuvant setting for GUCY2C-expressing gastrointestinal cancers (NCT04111172), results presented here are poised for translation to patients to determine the safety and activity of this combination (Ad5.F35 + Lm) targeting GUCY2C. Reflecting the superior quantity, quality, persistence, and antitumor efficacy of vaccine-induced CD8^+^ T-cell responses, all of which are important factors to protect colorectal cancer patients from recurrent and/or metastatic disease, this combination may produce significantly better clinical efficacy in patients than either single agent. Finally, this novel combination significantly enhanced antitumor efficacy, without toxicity towards normal GUCY2C-expressing tissues. Thus, this regimen may have superior GUCY2C-targeted efficacy without collateral safety risks to prevent recurrence in patients with colorectal cancer. Similarly, it may be efficacious in patients with gastric, esophageal, and pancreatic cancers, which often ectopically express GUCY2C^32^. Moreover, this may be an ideal vaccine strategy targeting other self or neo-antigens in cancers or chronic infectious diseases, such as HIV and tuberculosis, all settings for which there is a paucity of safe and effective vaccine platforms.

## Methods

### Vaccines

Replication-deficient chimeric serotype 5 adenovirus (Ad5.F35) expressing mouse GUCY2C_1-429_ fused to the influenza HA_107-119_ CD4^+^ T-cell epitope known as site 1 (S1) was previously described (indicated as “Ad-GUCY2C” throughout for simplicity)^19^. Viral vectors (control and GUCY2C-expressing Ad5.F35) were manufactured under Good Laboratory Practice (GLP) by the Baylor College of Medicine in the Cell and Gene Therapy Vector Development Lab and certified to be negative for replication-competent adenovirus, mycoplasma, and host cell DNA contamination.

The attenuated Lm strain containing deletions in virulence factors internalin B and actA, Δ*actA*Δ*inlB* Lm, was obtained from ATCC [*Listeria monocytogenes* (Murray et al.) Pirie (ATCC PTA-5562)] and served as the parental strain for all Lm vaccines utilized in this study. Recombinant Lm-GUCY2C^65^ and Lm-LacZ were generated by synthesizing DNA encoding mouse GUCY2C extracellular domain (GUCY2C_23-429_) or β-galactosidase (amino acids 618-1024), respectively, in-frame with a modified version of the first 100 amino acids of ActA protein (ActAN100*) and the Syn18x5 enhancer sequence under the control of the *actA* promoter^66^. The resulting sequence was cloned into the pPL2 integration vector and integrated into the Lm chromosome by direct conjugation from electroporated *E. coli* strain SM10^36^. Lm strains were grown in brain-heart infusion broth (Fisher Scientific) to an OD_600_ ~1, aliquoted, and stored at −80 °C^67^. On the day of experiments, aliquots were thawed, incubated at 37 °C for 60 min, washed 2× in PBS, and diluted to the desired concentration in PBS for vaccination.

### In vitro infections

The mouse macrophage cell line J774A.1 (ATCC) was cultured in DMEM supplemented with 10% FBS. J774A.1 cells were infected at a 10:1 multiplicity of infection with control or GUCY2C Lm. After a 1 h incubation at 37 °C, cells were washed 2× in PBS, resuspended in media containing 10 μg/mL gentamicin to eliminate free extracellular bacteria, and incubated an additional 5 h at 37 °C. For immunofluorescence studies, Lm was labeled prior to infection by incubation with 2 mM CellTracker Red CMPTPX dye for 10 min at 37 °C and GUCY2C protein was stained using the anti-GUCY2C monoclonal antibody MS20^68^ (2 μg/mL) followed by incubation with a peroxidase-conjugated 2° antibody (1:1000 Jackson ImmunoResearch # 115-035-062) for subsequent tyramide-FITC amplification. For western blot studies, the protein was extracted from cells using M-PER reagent (Pierce) supplemented with protease inhibitors. GUCY2C protein was stained using MS20^68^ (2 μg/mL) and p60 was stained using 1:5000 anti-p60 monoclonal antibody p6017 (AdipoGen #AG-20A-0023). Antibodies were detected with 1:25,000 2° antibody (Jackson ImmunoResearch # 115-035-062). Full blots are shown in Supplementary Fig. S7).

### Mice and immunizations

Eight-week-old male and female BALB/cJ mice were purchased from the Jackson Laboratory for experiments. Animal protocols were approved by the Thomas Jefferson University Institutional Animal Care and Use Committee (Protocol 01956). For adenovirus immunizations, mice received 10^10^ vp intramuscularly (i.m.) split into two 50 μL injections, one in each hind limb (unless otherwise indicated). For Lm immunizations, 5 × 10^6^ colony-forming units (CFU) were administered intravenously (i.v.) in a 100 μL suspension in PBS. For ELISpot experiments, empty Lm was used as the control vaccine and for tumor experiments, Lm-LacZ was used as control due to the ability of ActA protein to act as an adjuvant and augment anti-tumor responses^69^. For prime-boost immunizations, vaccines were delivered 21 days apart.

### Ad5-neutralizing immunity studies

Ad5 immunity was induced in BALB/cJ mice by intranasal exposure to 10^10^ vp Ad5-GFP once (to produce Ad5 NAb^lo^ titers) or twice (to produce Ad5 NAb^hi^ titers)^19^. Twenty-eight days after exposure(s), mice were bled, sera were collected, and mice were immunized with 10^11^ vp of Ad-GUCY2C (Ad5.F35-GUCY2C-S1) followed 21 days later by 5 × 10^6^ CFU of Lm-GUCY2C (control animals received Ad5.F35-Control and Lm-Control). Ad5 neutralizing antibody titers were quantified using neutralization of Ad5-GFP reporter vector infection of A549 cells with at least duplicate analyses per animal^19^. Dilutions of heat-inactivated serum samples were added to 96-well plates containing 10^5^ A549 cells (ATCC) and infected with 10^8^ vp of Ad5-CMV-eGFP (Baylor Vector Development Lab). Following a 41-hour incubation at 37 °C, eGFP fluorescence (490 nm excitation, 510 nm emission) was quantified using a POLARstar Optimate plate reader (BMG Labtech). Sample fluorescence was normalized to control wells containing cells and virus (0% neutralization) or wells containing cells alone (100% neutralization). Titers were quantified using non-linear regression as the serum dilution producing 50% neutralization (Prism v8, GraphPad Software).

### IFNγ ELISpot assay

ELISpot assays were performed using a mouse interferon-γ (IFNγ) single-color ELISpot kit (Cellular Technology Limited) according to the manufacturer’s protocol. Briefly, 96-well plates were coated with IFNγ capture antibody at 4 °C. After overnight incubation, plates were washed with PBS and splenocytes from immunized mice were plated in a 0.1% DMSO solution in CTL-TEST medium (Cellular Technology Limited) with or without 10 μg/mL GUCY2C_254-262_ peptide and incubated at 37 °C for 24 h in at least duplicate for each animal. For T-cell avidity studies, splenocytes were similarly analyzed using various concentrations of GUCY2C_254-262_ peptide (10 μg/mL to 3 pg/mL). Following overnight incubations, cells were removed, and development reagents were added to detect IFNγ-producing spot-forming cells (SFCs). The number of SFCs/well was calculated using the SmartCount and Autogate functions of an ImmunoSpot S6 Universal Analyzer (Cellular Technology Limited). GUCY2C-specific CD8^+^ T-cell responses were calculated by subtracting mean spot counts of 0.1% DMSO wells from peptide-pulsed wells.

### Intracellular cytokine staining

Splenocytes from immunized mice were plated in a 96-well plate at 10^6^/well in the presence of DMSO or 10 μg/mL GUCY2C _254-262_ peptide. Cells were incubated at 37 °C for 1 h, a protein transport inhibitor cocktail (eBioscience) was added, and splenocytes were incubated for an additional 5 h at 37 °C. Cells were then stained with LIVE/DEAD Fixable Aqua Dead Cell Stain Kit (Invitrogen), anti-CD8-PerCP-Cy5.5 (clone 53-6.7; BD Biosciences # 551162; 1:200 dilution), and anti-CD19-BV510 (clone 6D5; Biolegend # 115545; 1:200 dilution). A BD Cytofix/Cytoperm Kit (BD Biosciences) was used for permeabilization and intracellular cytokine staining using anti-IFNγ-PE-CF594 (clone XMG1.2; BD Biosciences # 562333; 1:200 dilution), anti-TNFα-PE-Cy7 (clone MP6-XT22, BD Biosciences # 561041; 1:200 dilution), and anti-MIP1α-APC (clone 39624; R&D Systems # IC450A; 1:200 dilution). Cells were fixed in 2% paraformaldehyde and analyzed on a BD LSR II flow cytometer. Analyses were performed using FlowJo software (TreeStar).

### Tumor studies

The BALB/cJ CT26 colorectal cancer cell line expressing GUCY2C and luciferase^19^ was used for in vivo tumor studies. For most studies, six days after final immunizations, mice received 5 × 10^5^ CT26 cells via intravenous (i.v.) tail vein injection. However, for memory studies, tumor challenges occurred 63 days after the final immunization. Tumor burden was quantified by subcutaneous injection of 3.75 mg of D-luciferin potassium salt (Gold Biotechnologies) in PBS followed by an eight-minute incubation and imaging with a ten-second exposure using a Caliper IVIS Lumina XR imaging station (PerkinElmer). Total radiance (photons/second) was measured using Living Image In Vivo Imaging Software (PerkinElmer).

### Safety studies

#### Survival, body weight, and histopathology assessment (Fig. 5)

BALB/cJ mice were primed with 10^10^ vp of Ad5.F35-GUCY2C-S1 i.m. split into two 50 μL injections, one in each hind limb, on day 0. Mice were then boosted with 2 i.v. administrations of 5 × 10^6^ CFU of Lm-GUCY2C in a 100 μL suspension in PBS at 21-day intervals (i.e., boosted on days 21 and 42). Control animals received PBS instead of Ad or Lm immunizations. Animals were monitored for adverse events and body weight and on days 49 and 72, designated animals were sacrificed and indicated tissues were harvested for histopathological analysis by a blinded pathologist (pathology evaluation was performed by IDEXX BioAnalytics).

#### Blood chemistry and cytokine analyses (Supplementary Figs. S5 and S6)

BALB/cJ mice were primed with 10^10^ vp of Ad5.F35-GUCY2C-S1 or Ad5.F35-Control and boosted with i.v. administration of 5 × 10^6^ CFU of Lm-GUCY2C or Lm-Control in a 100 μL suspension in PBS at a 21-day interval. Clinical chemistry analyses included an additional cohort of animals that received only PBS for all administrations. Animals were euthanized 6 days after Lm administration and plasma was collected for multiplex cytokine quantification by Eve Technologies and clinical chemistry profiling by Charles River Laboratories.

### Reporting summary

Further information on research design is available in the Nature Research Reporting Summary linked to this article.

## Supplementary information

Supplementary Material

REPORTING SUMMARY

## Data Availability

Statistical analyses were carried out using GraphPad Prism v9. The datasets generated and analyzed during this study are available from the corresponding author. Biological materials are available following the Material Transfer Agreement execution from the corresponding author.
