# Supplementary material for: Chimeric adenoviral (Ad5.F35) and listeria vector prime-boost immunization is safe and effective for cancer immunotherapy
Source: NPJ Vaccines. 2022 Jun 23;7:61. doi: 10.1038/s41541-022-00483-z (PMC9226178; doi:10.1038/s41541-022-00483-z)
Supplement: Supplementary file 1 — Supplementary Material [file 41541_2022_483_MOESM1_ESM.pdf]

<SUPPLEMENTARY INFORMATION FILE>

Prime-Boost Immunization with Chimeric Adenoviral (Ad5.F35) and *Listeria* Vectors is a Safe and Effective Strategy for Cancer Immunotherapy

John C. Flickinger Jr, Ross E. Staudt, Jagmohan Singh, Robert Carlson, Joshua R. Barton, Trevor R. Baybutt, Jeffrey A. Rappaport, Alicja Zalewski, Amanda Pattison, Scott A. Waldman, Adam E. Snook

TABLE OF CONTENTS

Supplementary Figure 1. Ad-GUCY2C + Lm-GUCY2C is ineffective in a therapeutic model of metastatic colorectal cancer. 2

Supplementary Figure 2. Lm-GUCY2C boosting enhances GUCY2C-specific memory CD8$^+$ T-cell responses. 3

Supplementary Figure 3. Lm-GUCY2C boosts GUCY2C-specific CD8$^+$ T-cells generated long after initial priming vaccination. 4

Supplementary Figure 4. Prior Lm exposure does not reduce GUCY2C-specific CD8$^+$ T-cell expansion by Lm-GUCY2C boosting. 5

Supplementary Figure 5. Blood cytokine responses to Ad-GUCY2C + Lm-GUCY2C immunization. 6

Supplementary Figure 6. Clinical chemistry profiles following Ad-GUCY2C + Lm-GUCY2C immunization. 7

Supplementary Figure 7. Uncropped western blot images. 8

Supplementary Figure 8. Representative intracellular cytokine staining for polyfunctionality analysis. 9

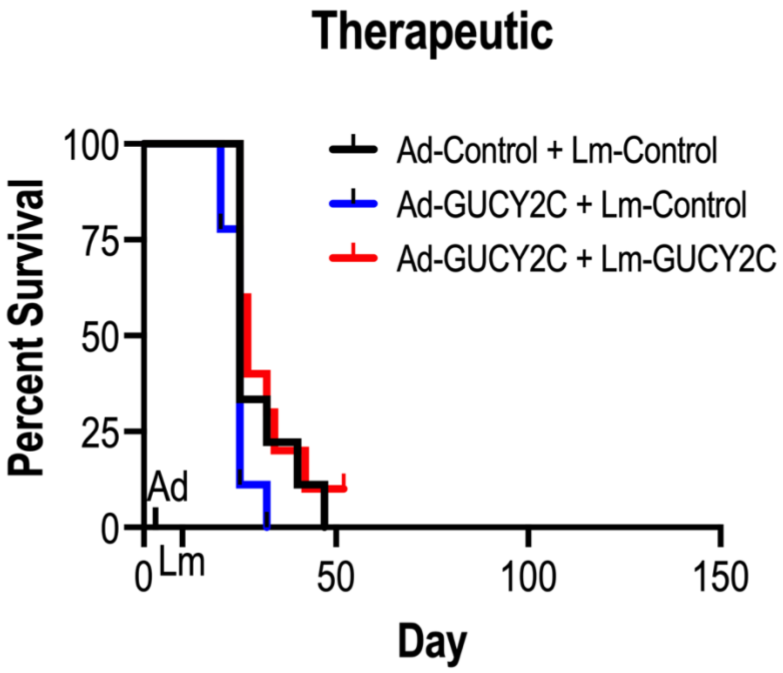

Supplementary Figure 1. Ad-GUCY2C + Lm-GUCY2C is ineffective in a therapeutic model of metastatic colorectal cancer. Metastatic colorectal cancer was established by i.v. administration of $1x10^5$ CT26 colorectal cancer cells to BALB/cJ mice (n = 9-10/group). Mice were immunized as in **Fig. 3**, except an abbreviated scheduled was used: primed on day 3 with Ad5.F35 (Ad) vaccines and boosted 7 days later with Lm vaccines. Survival was monitored throughout the experiment and analyzed by the Mantel-Cox log rank test with all immunized groups compared to control immunization using the Bonferroni method to correct for multiple comparisons. No significant differences were observed.

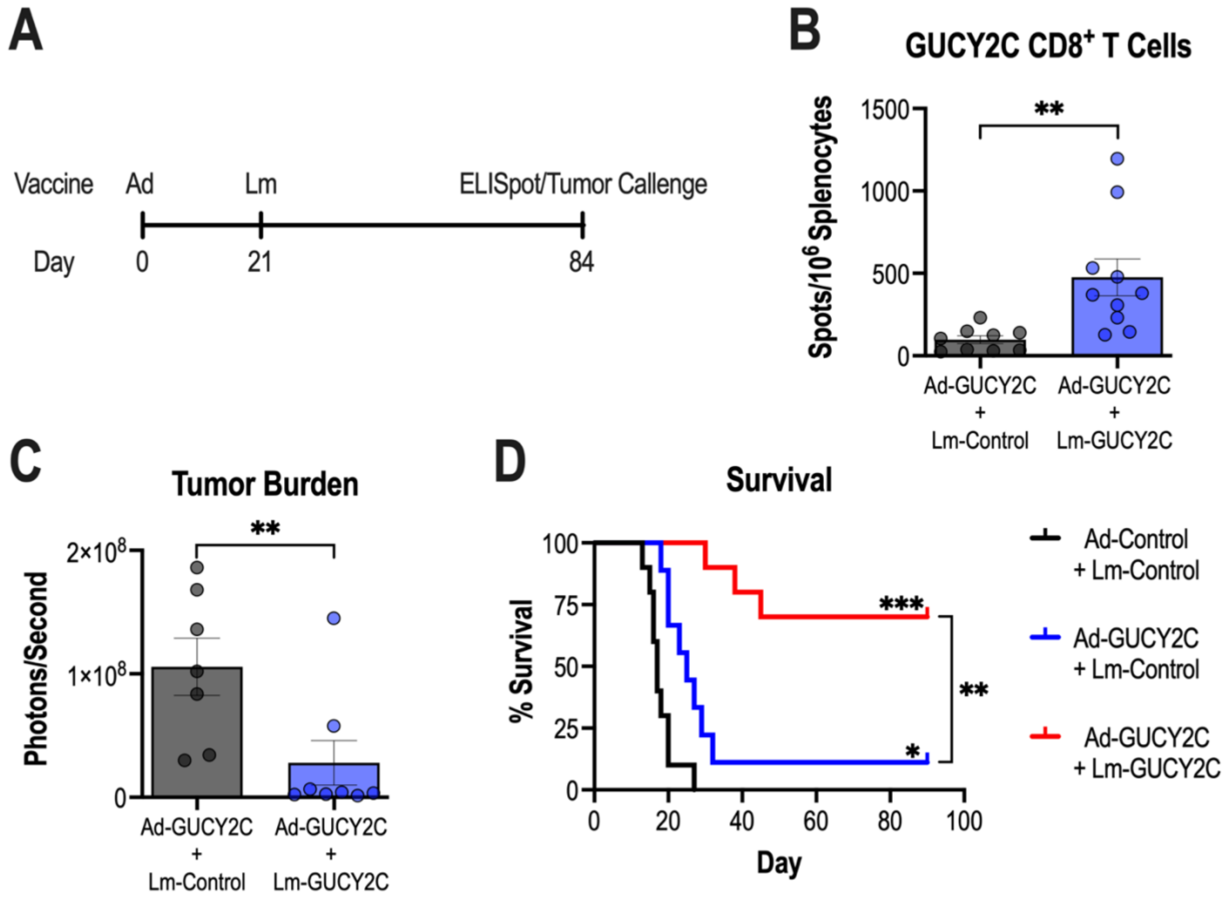

Supplementary Figure 2. Lm-GUCY2C boosting enhances GUCY2C-specific memory CD8$^+$ T-cell responses. A-D BALB/cJ mice were immunized with 10^{10} vp of control or GUCY2C adenovirus (Ad5) on day 0 followed by boosting with $5x10^6$ CFU of control or GUCY2C Lm on day 21. Sixty-three days later, mice were euthanized to quantify GUCY2C-specific CD8$^+$ T-cell responses (**A**) or challenged with $5x10^5$ CT26 colorectal cancer cells expressing GUCY2C and firefly luciferase (**C-D**). Tumor burden was quantified on day 20 (**C**) and survival was monitored throughout the experiment (**D**). GUCY2C-specific CD8$^+$ T-cell counts (**B**) and tumor-burden (**C**) were analyzed by T-test and survival (**D**) was analyzed by the Mantel-Cox log rank test (multiple comparisons were corrected with the Bonferroni method). In **D**, significance compared to control is indicated on the line and compared between GUCY2C vaccine groups as indicated. Error bars indicate mean +/- SEM.

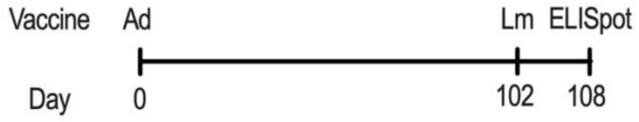

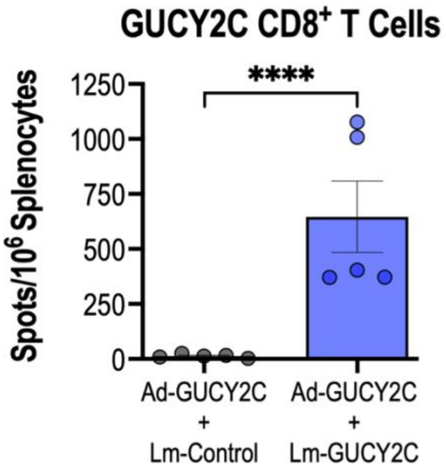

Supplementary Figure 3. Lm-GUCY2C boosts GUCY2C-specific CD8$^+$ T-cells generated long after initial priming vaccination. BALB/cJ mice were immunized intramuscularly with 10^{10} vp of Ad-GUCY2C, boosted 102 days later with $5x10^6$ CFU of control or GUCY2C Lm intravenously. Six days later, GUCY2C-specific CD8$^+$ T-cells counts were quantified by IFNγ-ELISpot. GUCY2C-specific CD8$^+$ T-cell counts were analyzed by T-test. Error bars indicate mean +/- SEM.

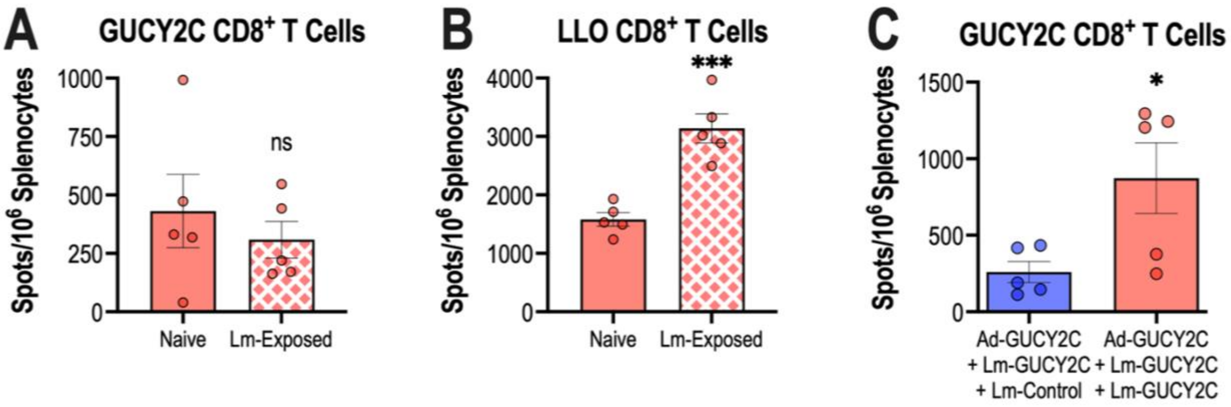

Supplementary Figure 4. Prior Lm exposure does not reduce GUCY2C-specific CD8$^+$ T-cell expansion by Lm-GUCY2C boosting. A-B BALB/cJ mice were exposed to control Lm by intravenous administration of 5×10^6 CFU or left naïve (vehicle exposure) on day 0. Mice were immunized on day 14 with 10^{10} vp of Ad-GUCY2C intramuscularly and boosted on day 35 with 5×10^6 CFU of Lm-GUCY2C intravenously. Six days later, mice were euthanized and GUCY2C-specific (**A**) and LLO-specific (**B**) CD8$^+$ T-cell counts were quantified by IFNγ-ELISpot. **C** BALB/cJ mice were immunized intramuscularly with 10^{10} vp of Ad-GUCY2C on day 0, boosted with 5×10^6 CFU of Lm-GUCY2C on day 21, and on day 42, mice were boosted a second time with 5×10^6 CFU of control or GUCY2C Lm. GUCY2C-specific CD8$^+$ T-cells were quantified by IFNγ-ELISpot 6 days after final immunization. T-cell counts were analyzed by T-test. Error bars indicate mean +/- SEM.

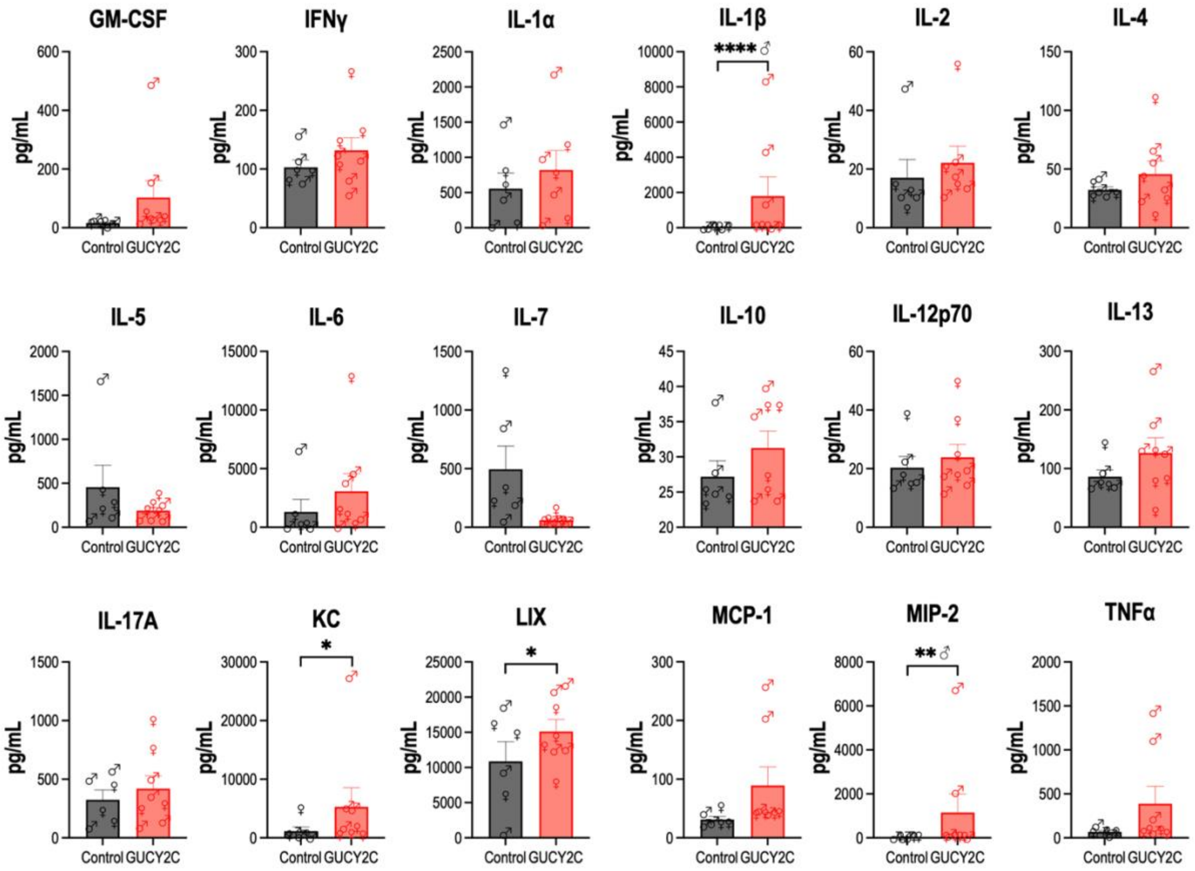

Supplementary Figure 5. Blood cytokine responses to Ad-GUCY2C + Lm-GUCY2C immunization. BALB/cJ mice were primed with 10^{10} vp of control or GUCY2C Ad5.F35 i.m. and boosted with $5x10^6$ CFU of control or GUCY2C Lm i.v. at a 21-day interval. Plasma was collected 6 days later, and cytokines were quantified (Charles River). Statistics indicate comparisons by two-way ANOVA, with both sexes analyzed together. IL1-β and MIP-2 were significantly different only in males. Symbols indicate male (♂) and female (♀) mice. Error bars indicate mean +/- SEM.

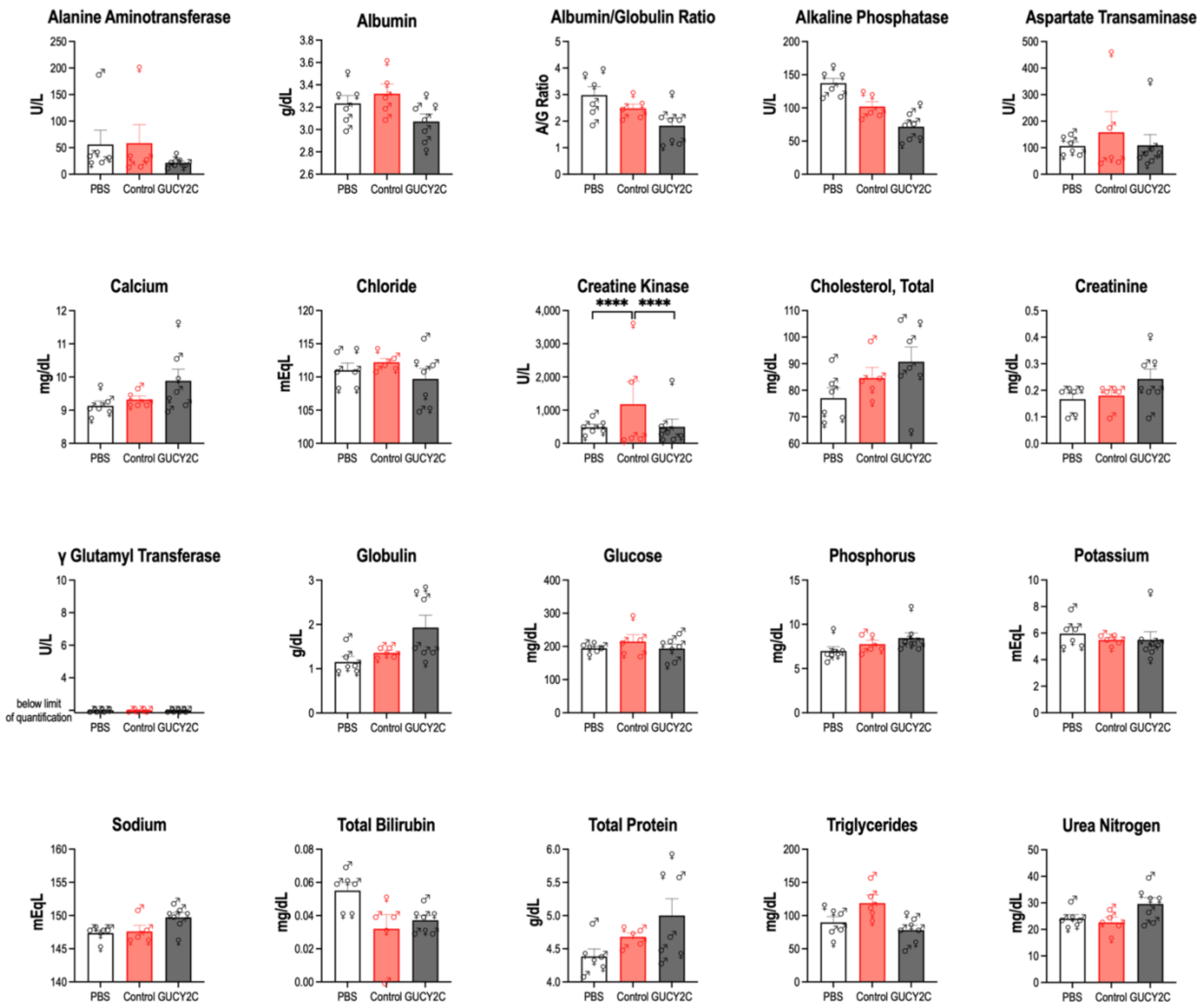

Supplementary Figure 6. Clinical chemistry profiles following Ad-GUCY2C + Lm-GUCY2C immunization. BALB/cJ mice received only PBS or were primed with 10^{10} vp of control or GUCY2C Ad5.F35 i.m. and boosted with $5x10^6$ CFU of control or GUCY2C Lm i.v. at a 21-day interval. Plasma was collected 6 days later, and analytes were quantified (Eve Technologies). Statistics indicate comparisons by two-way ANOVA, with both sexes analyzed together. Symbols indicate male (♂) and female (♀) mice. Error bars indicate mean +/- SEM.

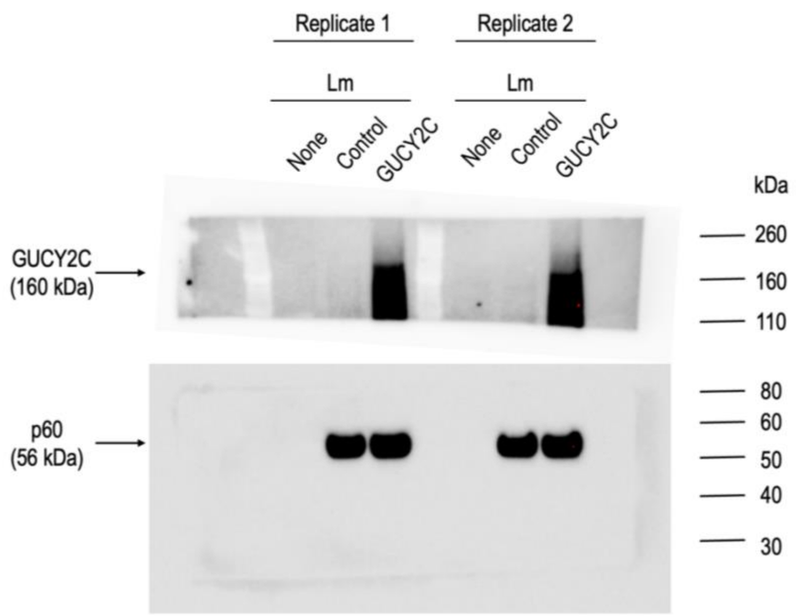

Supplementary Figure 7. Uncropped western blot images. Uncropped western blot images of replicate samples pertaining to cropped images shown in **Fig. 1C**, as indicated above.

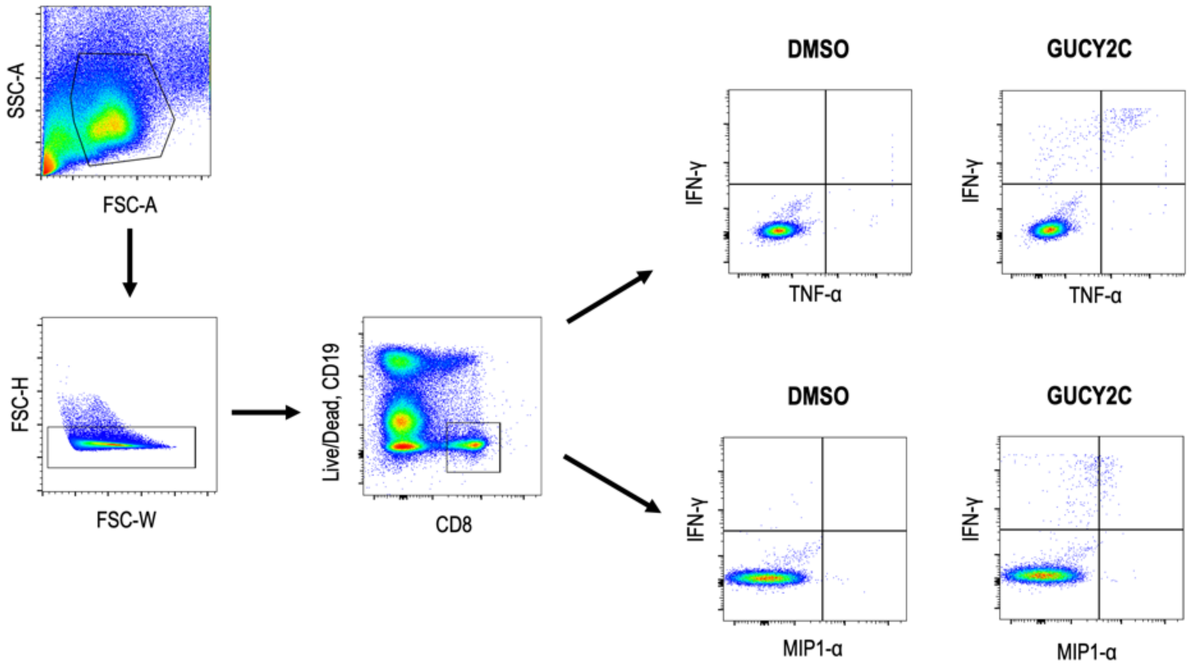

Supplementary Figure 8. Representative intracellular cytokine staining for polyfunctionality analysis. Lymphocytes were gated based on SSC-A and FSC-A profiles with doublets excluded based on FSC-H vs. FSC-W profiles. Dead cells and CD19$^+$ B cells were excluded by staining with LIVE/DEAD Aqua Blue and anti-CD19-BV510 antibody. Live/CD19$^-$CD8$^+$ cells were gated and assessed for IFNγ, TNFα, and/or MIP1α staining using FlowJo Boolean gates (IFNγ$^+$TNFα$^-$MIP1a$^-$, IFNγ$^+$TNFα$^+$MIP1a$^-$, etc). A representative immunized mouse stimulated with DMSO or GUCY2C$_{254\text{-}262}$ peptide is shown, demonstrating specific detection of GUCY2C-reactive CD8$^+$ T cells. Single, double, and triple cytokine population frequencies are depicted in **Fig. 4B-D**.
