# Supplementary material for: Chimeric adenoviral (Ad5.F35) and listeria vector prime-boost immunization is safe and effective for cancer immunotherapy
Source: NPJ Vaccines. 2022 Jun 23;7:61. doi: 10.1038/s41541-022-00483-z (PMC9226178; doi:10.1038/s41541-022-00483-z)
Supplement: Supplementary file 2 — REPORTING SUMMARY [file 41541_2022_483_MOESM2_ESM.pdf]

nature portfolio

Corresponding author(s): Adam Snook

Last updated by author(s): Mar 21, 2022

Reporting Summary

Nature Portfolio wishes to improve the reproducibility of the work that we publish. This form provides structure for consistency and transparency in reporting. For further information on Nature Portfolio policies, see our Editorial Policies and the Editorial Policy Checklist.

Statistics

For all statistical analyses, confirm that the following items are present in the figure legend, table legend, main text, or Methods section.

n/a	Confirmed	
☐	☒	The exact sample size (n) for each experimental group/condition, given as a discrete number and unit of measurement
☐	☒	A statement on whether measurements were taken from distinct samples or whether the same sample was measured repeatedly
☐	☒	The statistical test(s) used AND whether they are one- or two-sided<br>*Only common tests should be described solely by name; describe more complex techniques in the Methods section.*
☒	☐	A description of all covariates tested
☐	☒	A description of any assumptions or corrections, such as tests of normality and adjustment for multiple comparisons
☐	☒	A full description of the statistical parameters including central tendency (e.g. means) or other basic estimates (e.g. regression coefficient) AND variation (e.g. standard deviation) or associated estimates of uncertainty (e.g. confidence intervals)
☐	☒	For null hypothesis testing, the test statistic (e.g. F, t, r) with confidence intervals, effect sizes, degrees of freedom and P value noted<br>*Give P values as exact values whenever suitable.*
☒	☐	For Bayesian analysis, information on the choice of priors and Markov chain Monte Carlo settings
☒	☐	For hierarchical and complex designs, identification of the appropriate level for tests and full reporting of outcomes
☒	☐	Estimates of effect sizes (e.g. Cohen's d, Pearson's r), indicating how they were calculated

Our web collection on statistics for biologists contains articles on many of the points above.

Software and code

Policy information about availability of computer code

Data collection	N/A
Data analysis	GraphPad Prism v9

For manuscripts utilizing custom algorithms or software that are central to the research but not yet described in published literature, software must be made available to editors and reviewers. We strongly encourage code deposition in a community repository (e.g. GitHub). See the Nature Portfolio guidelines for submitting code & software for further information.

Data

Policy information about availability of data

All manuscripts must include a data availability statement. This statement should provide the following information, where applicable:

- Accession codes, unique identifiers, or web links for publicly available datasets
- A description of any restrictions on data availability
- For clinical datasets or third party data, please ensure that the statement adheres to our policy

The datasets generated and analyzed during this study are available from the corresponding author.

Field-specific reporting

Please select the one below that is the best fit for your research. If you are not sure, read the appropriate sections before making your selection.

☒ Life sciences ☐ Behavioural & social sciences ☐ Ecological, evolutionary & environmental sciences

For a reference copy of the document with all sections, see nature.com/documents/nr-reporting-summary-flat.pdf

Life sciences study design

All studies must disclose on these points even when the disclosure is negative.

Sample size	Sample sizes were determined by power calucations
Data exclusions	There were no data exclusions
Replication	All animal data are presented for in vivo and ex vivo (ex, ELISpot) studies. In vitro studies were replicated at least once.
Randomization	Animals were randomized to cohorts.
Blinding	Animals were assigned a number during randomization and investigators were blinded to allocation during data collection and analysis.

Reporting for specific materials, systems and methods

We require information from authors about some types of materials, experimental systems and methods used in many studies. Here, indicate whether each material, system or method listed is relevant to your study. If you are not sure if a list item applies to your research, read the appropriate section before selecting a response.

Materials & experimental systems

n/a	Involved in the study	
☐	☒	Antibodies
☐	☒	Eukaryotic cell lines
☒	☐	Palaeontology and archaeology
☐	☒	Animals and other organisms
☒	☐	Human research participants
☒	☐	Clinical data
☒	☐	Dual use research of concern

Methods

n/a	Involved in the study	
☒	☐	ChIP-seq
☐	☒	Flow cytometry
☒	☐	MRI-based neuroimaging

Antibodies

Antibodies used

1. anti-GUCY2C antibody MS20 (in-house production)
2. anti-p60 monoclonal antibody (clone p6017; AdipoGen)
3. anti-CD8-PerCP-Cy5.5 (clone 53-6.7; BD Biosciences)
4. anti-CD19-BV510 (clone 6D5; Biolegend)
5. anti-IFNγ-PE-CF594 (clone XMG1.2; BD Biosciences)
6. anti-TNFα-PE-Cy7 (clone MP6-XT22, BD Biosciences)
7. anti-MIP1α-APC (clone 39624; R&D Systems)

Validation

1. anti-GUCY2C antibody MS20 (in-house production) validated in Marszalowicz, G. P., et al. Oncotarget 5, 9460–9471 (2014) and routinely re-validated by western blot on GUCY2C+/+ and GUCY2C-/- tissues.
2. anti-p60 monoclonal antibody p6017 (AdipoGen) validated on negative-control and recombinant listeria monocytogenes cultures.
3.-7. The anti-CD8, anti-CD19, anti-IFNγ, anti-TNFα, and anti-MIP1α clones have been used and validated extensively. References for each are available on the BD Biosciences, Biolegend, and R&D Systems websites.

Eukaryotic cell lines

Policy information about cell lines

Cell line source(s)	CT26 and J774A.1 cells were from ATCC
Authentication	STR Profiling was employed for authentication.
Mycoplasma contamination	Negative mycoplasma contamination was routinely confirmed.

Commonly misidentified lines (See ICLAC register)	No commonly misidentified cell lines were used.

Animals and other organisms

Policy information about studies involving animals; ARRIVE guidelines recommended for reporting animal research

Laboratory animals	Male and female BALB/cJ mice from Jax Laboratory, 8-12 weeks of age were used.
Wild animals	No wild animals were used.
Field-collected samples	No field-collected samples were used.
Ethics oversight	The Thomas Jefferson University Institutional Animal Care and Use Committeee (IACUC) provided oversight.

Note that full information on the approval of the study protocol must also be provided in the manuscript.

Flow Cytometry

Plots

Confirm that:

- [ ] The axis labels state the marker and fluorochrome used (e.g. CD4-FITC).
- [ ] The axis scales are clearly visible. Include numbers along axes only for bottom left plot of group (a 'group' is an analysis of identical markers).
- [ ] All plots are contour plots with outliers or pseudocolor plots.
- [x] A numerical value for number of cells or percentage (with statistics) is provided.

Methodology

Sample preparation	Splenocytes were collected from immunized animals by mechanical discruption and red blood cell lysis. Samples were plated in a 96-well plate at 1e6/well in the presence of DMSO or peptide. Cells were incubated at 37°C for 1h, protein transport inhibitor cocktail (eBioscience) was added, and splenocytes were incubated an additional 5 hours at 37°C. Cells were then stained with LIVE/DEAD Fixable Aqua Dead Cell Stain Kit (Invitrogen), anti-CD8-PerCP-Cy5.5 (clone 53-6.7; BD Biosciences), and anti-CD19-BV510 (clone 6D5; Biolegend). A BD Cytofix/Cytoperm Kit (BD Biosciences) was used for permeabilization and intracellular cytokine staining using anti-IFNγ-PE-CF594 (clone XMG1.2; BD Biosciences), anti-TNFα-PE-Cy7 (clone MP6-XT22, BD Biosciences), and anti-MIP1α-APC (clone 39624; R&D Systems). Cells were fixed in 2% paraformaldehyde.
Instrument	BD LSR II flow cytometer
Software	Analyses were performed using FlowJo software (TreeStar)
Cell population abundance	No sorting was performed.
Gating strategy	Gates were created based on FMO samples. The full gating strategy for a representative sample is provided in Supplementary Fig. S8.

- [x] Tick this box to confirm that a figure exemplifying the gating strategy is provided in the Supplementary Information.
